# Effectiveness and safety of eribulin in Japanese patients with HER2-negative, advanced breast cancer: a 2-year post-marketing observational study in a real-world setting

**DOI:** 10.1007/s10637-019-00890-5

**Published:** 2020-01-16

**Authors:** Kenichi Inoue, Masato Takahashi, Hirofumi Mukai, Takashi Yamanaka, Chiyomi Egawa, Yukinori Sakata, Hiroki Ikezawa, Toshiyuki Matsuoka, Junji Tsurutani

**Affiliations:** 1 Division of Breast Oncology, Saitama Cancer Center, Saitama, Japan; 2 Department of Breast Surgery, National Hospital Organization Hokkaido Cancer Center, Hokkaido, Japan; 3 Division of Breast and Medical Oncology, National Cancer Center Hospital East, Chiba, Japan; 4 Department of Breast and Endocrine Surgery, Kanagawa Cancer Center, Kanagawa, Japan; 5 Department of Breast Surgery, Kansai Rosai Hospital, Hyogo, Japan; 6 Clinical Planning and Development Department, Eisai Co., Ltd., Tokyo, Japan; 7 Advanced Cancer Translational Research Institute, Showa University, Tokyo, Japan; 8 Department of Medical Oncology, Kindai University, Osaka, Japan

**Keywords:** Eribulin, Post-marketing study, Japan, Overall survival

## Abstract

*Background* Data on eribulin as the first- or second-line treatment in a clinical setting, especially the overall survival (OS) of patients, are scarce. Therefore, we assessed the effectiveness and safety of eribulin as the first-, second-, and third- or later-line treatments in patients with human epidermal growth factor receptor 2 (HER2)-negative advanced breast cancer in Japan. *Methods* This multicenter, prospective, post-marketing, observational study enrolled patients from September 2014 to February 2016 in Japan and followed them for 2 years. Patients were categorized by eribulin use into the first-, second-, and third- or later-line treatment groups. *Results* Of 651 registered patients, 637 patients were included in the safety and effectiveness analysis. In all, first-, second-, and third or later-line treatment groups, median OS (95% confidence interval) were 15.6 (13.8–17.6), 22.8 (17.3–31.0), 16.3 (12.4–19.9), and 12.6 (11.2–15.1) months and time to treatment failure (TTF) (95% confidence interval) were 4.2 (3.7–4.4), 5.2 (3.7–5.9), 4.2 (3.7–5.1), and 3.8 (3.5–4.2) months, respectively. Prolonged TTF was associated with complications of diabetes and the development of peripheral neuropathy after eribulin treatment, according to multivariate Cox regression analysis. Grade ≥ 3 adverse drug reactions (ADRs) were reported in 61.7% of the patients. Neutropenia (49.5%) was the most common grade ≥ 3 ADR in all groups. *Conclusions* The effectiveness and safety results of eribulin as the first- or second-line treatment were favorable. Thus, these suggest eribulin may be a first-line treatment candidate for patients with HER2-negative advanced breast cancer in Japan.

## Introduction

Breast cancer in Japan is one of the cancers with the highest incidence [1]. Although breast cancer treatments have progressed, the majority of cases with metastatic breast cancer remains incurable with a 5-year relative survival rate of 35.2% for women with stage IV breast cancer in Japan [2]. Hence, there is an urgent need to develop metastatic breast cancer treatment that prolongs patient’s survival, alleviates symptoms, and improves the quality of life (QoL) [3]. Currently, the Japanese Breast Cancer Society recommends anthracyclines, taxanes, and S-1 as the first-line treatment for patients with metastatic or recurrent epidermal growth factor receptor 2 (HER2)-negative breast cancer [4]. However, a single agent for metastatic or recurrent HER2-negative breast cancer seems to be limited suggesting the need for alternative treatment options.

Eribulin mesylate (Halaven®, Eisai Co. Ltd., Tokyo, Japan) is a non-taxane microtubule dynamics inhibitor that was approved for the treatment of advanced breast cancer in Japan in 2011. Eribulin exerts antitumor activity through a unique mechanism of action which is unlike that of other chemotherapeutic agents such as paclitaxel and docetaxel. Eribulin was granted global approval for patients as the third- or later-line treatment, mainly because of the prolonged overall survival (OS) result shown in a phase 3 EMBRACE study [5].

The efficacy and safety of eribulin as the first- or second-line treatment was not explored in randomized controlled trials. However, some clinical trials (mainly phase 2) and a real-world study reported the efficacy and safety of eribulin as a first- or second-line treatment [6–15]. Jacot et al. reported the activities of eribulin among metastatic breast cancer patients in a multicenter national observational Epidemiological Strategy and Medical Economic (ESME) program in a real-world setting [14]. They concluded that patients with HER2-negative metastatic breast cancer who received eribulin as a second-, third-, or fourth-chemotherapy-line presented a significantly better progression-free survival and OS than those receiving other chemotherapy agents. However, no first-line treatment results were presented in these studies. Furthermore, the limitations of these studies were small number of patients and /or no OS results.

Additionally, a post-marketing study in Japan reported the effectiveness and safety of eribulin in a clinical setting [16]. However, in this previous study, OS was not assessed and data on the effectiveness and safety of eribulin as a first- or second-line treatment was only included for <10% of patients who used eribulin as a first- or second-line treatment [16]. Thus, data on eribulin as a first- or second-line treatment in a clinical setting, particularly on OS, are scarce. Data on the effectiveness and safety of eribulin as a first- or second-line treatment was collected in a real-world setting only in Japan. Thus, we conducted a 2-year post-marketing study in patients with HER2-negative inoperable or recurrent breast cancer in a clinical setting in Japan to assess the effectiveness (OS) and safety of eribulin including in patients using eribulin as a first- or second-line treatment. We previously reported the interim-analysis results of this study which focused on peripheral neuropathy as one of the main aims of this study [17]. Herein, we report the final analysis results to assess the effectiveness and safety of eribulin as a first-, second-, and third or later-line treatment in patients with HER2-negative advanced breast cancer in Japan.

## Patients and methods

### Study design

This was a multicenter, prospective, post-marketing, observational study conducted in Japan (ClinicalTrials.gov: NCT02371174). Patients were enrolled from September 2014 to February 2016 and followed-up for 2 years. Details of the study are available elsewhere [17].

Eisai Co., Ltd. reviewed the scientific and ethical validity of the study design. This study was conducted in accordance with the Declaration of Helsinki and Japanese Good Post-Marketing Study Practice (GPSP), an authorized standard for post-marketing surveillance. GPSP does not require approval from the institutional review boards of each institution or informed consent from the participating patients. However, in practice, some institutions may have obtained approval or informed consent when deemed necessary. Personal data related to this study were managed in compliance with the privacy protection laws in Japan.

### Patients

Eribulin-naïve patients with HER2-negative inoperable or recurrent breast cancer who received eribulin as first/second-line or as third/later-line chemotherapy were recruited in each institution in approximately equal numbers (1:1 ratio). Pre- and post-operative chemotherapy, hormone therapy, antibody therapy, immunotherapy, and local radiation therapy were not included in “Previous chemotherapy regimens”. Exclusion criteria were patients with severe bone-marrow suppression defined as a neutrophil count of <1,000/mm^3^ or platelet count <75,000/mm^3^, patients with a history of hypersensitivity to the eribulin components, and pregnant or possibly pregnant patients.

### Eribulin administration

Patients generally received eribulin intravenously at a dose of 1.4 mg/m^2^ over 2 to 5 min on day 1 (initiation of eribulin treatment; baseline) and day 8 of a 21-day cycle as indicated. For some patients (such as those with hepatic dysfunction), the starting dose of eribulin was reduced (1.1 mg/m^2^), depending on the patient’s condition to prevent toxicity.

### Data collection

Patients were registered at a central registration system, and data were collected using registration and case report forms (CRFs). CRFs were collected after the following observation periods: 1) baseline to 6 months; 2) >6 months after baseline to 1 year; 3) >1 year after baseline to 2 years. For patients who discontinued treatment in this study, patient survival outcomes (alive/dead) were collected until the end of the 2-year period from the first eribulin administration date.

Collected data were baseline characteristics (e.g., age and gender), treatment history (e.g., history of radiotherapy), eribulin administration (e.g., administered dose and cycles), treatment status of eribulin (i.e., treatment continued/discontinued and reason for discontinuation), patient survival outcome (alive/dead), laboratory test results, and adverse events.

### Assessment and definition

Effectiveness was assessed by OS, time to treatment failure (TTF), and factors affecting TTF. OS was defined as the time from the first eribulin dose administration until all-cause death or the last date the patient was known to be alive (censored). TTF was defined as the time from the first eribulin dose administration until the date of treatment discontinuation from any cause (e.g., death, documentation of disease progression, adverse events, or patient’s request).

Safety was assessed by adverse drug reactions (ADRs) and the number of patients who discontinued eribulin treatment due to ADRs. The severity of ADRs was assessed based on the Japanese version of the Common Terminology Criteria for Adverse Events (CTCAE), version 4.0. ADRs were classified according to the Japanese version of the Medical Dictionary for Regulatory Activities (version 21.1).

### Statistical analysis

Patients were categorized by their use of eribulin into first-, second-, and third or later-line treatment. Baseline characteristics, eribulin treatment status, all grade ADRs, grade ≥ 3 ADRs, and the number of patients who discontinued eribulin due to ADRs were summarized descriptively. Using the Kaplan-Meier method, median OS and TTF (95% confidence interval [CI]) in months were estimated. The survival rate and percentage of patients who continued eribulin for 1 and 2 years were calculated.

To assess factors affecting TTF, we conducted univariate and multivariate Cox regression analyses. First, the hazard ratio (HR) and 95% CI were calculated for each factor. Subsequently, a stepwise method was used for the multivariate analysis with selection criteria of *p* < 0.20. All statistical factors with *p* < 0.05 were considered statistically significant. The factors included in the multivariate Cox regression analysis were those influencing the development of peripheral neuropathy after eribulin treatment (i.e., new onset of peripheral neuropathy or worsening of existing peripheral neuropathy from baseline), including visceral metastasis, triple-negative, age, menopause, Eastern Cooperative Oncology Group Performance Status (ECOG PS), history of radiotherapy, liver metastasis, lung metastasis, bone metastasis, eribulin start dose, complication of diabetes, complication of liver dysfunction, complication of renal dysfunction, complication of hypertension, body mass index (BMI), development of peripheral neuropathy after previous chemotherapy, number of previous chemotherapy regimens, drug for peripheral neuropathy prevention during eribulin treatment, hemoglobin levels at baseline, aspartate aminotransferase (AST) at baseline, and creatinine at baseline.

All analyses were performed using SAS software version 9.4 (SAS Institute, Inc., Cary, North Carolina).

## Results

### Patients

In this prospective observational study, 651 patients were registered from 182 institutions, and of these, 637 patients were included in the safety and effectiveness analysis.

The baseline characteristics of the patients are summarized in Table 1. In each treatment line, 142 (22.3%), 177 (27.8%), 151 (23.7%), 75 (11.8%), 57 (8.9%), and 34 (5.3%) patients used eribulin as first-, second-, third-, fourth-, and fifth or later-line treatment at baseline, respectively. The mean age (± standard deviation [SD]) of patients in each line was similar: 59.4 ± 11.5, 59.6 ± 10.7, and 59.6 ± 10.9 years in the first-, second-, and third or later-line treatment group, respectively. Baseline characteristics in each group did not largely differ, except for ECOG PS, which ranged from 0 to 3 in each of the three treatment groups: 69.0%–1.4%, 63.3%–0.6%, and 47.0%–0.6%, respectively.

**Table 1:** Baseline characteristics

	All^a)^ *n* = 637		First-line *n* = 142		Second-line *n* = 177		Third or later-line *n* = 317	
Gender, n (%)								
Female	634	(99.5)	142	(100)	174	(98.3)	317	(100)
Age (years)								
Mean ± SD	59.5 ± 11.0		59.4 ± 11.5		59.6 ± 10.7		59.6 ± 10.9	
Range (min–max)	30–85		34–82		30–83		32–85	
Menopause status, n (%)								
Pre	85	(13.4)	25	(17.6)	21	(12.1)	39	(12.3)
Post	509	(80.3)	113	(79.6)	138	(79.3)	258	(81.4)
Unknown	40	(6.3)	4	(2.8)	15	(8.6)	20	(6.3)
ECOG PS, n (%)								
0	360	(56.5)	98	(69.0)	112	(63.3)	149	(47.0)
1	234	(36.7)	39	(27.5)	53	(29.9)	142	(44.8)
2	38	(6.0)	3	(2.1)	11	(6.2)	24	(7.6)
3	5	(0.8)	2	(1.4)	1	(0.6)	2	(0.6)
Hormone receptor status, n (%)								
Positive	462	(72.5)	95	(66.9)	124	(70.1)	242	(76.3)
Negative	157	(24.6)	44	(31.0)	46	(26.0)	67	(21.1)
Unknown	18	(2.8)	3	(2.1)	7	(4.0)	8	(2.5)
Triple negative (HER2-negative, ER-negative, PgR-negative), n (%)								
No	462	(72.5)	95	(66.9)	124	(70.1)	242	(76.3)
Yes	157	(24.6)	44	(31.0)	46	(26.0)	67	(21.1)
Unknown	18	(2.8)	3	(2.1)	7	(4.0)	8	(2.5)
History of radiotherapy, n (%)								
No	494	(77.6)	98	(69.0)	139	(78.5)	256	(80.8)
Yes	135	(21.2)	43	(30.3)	34	(19.2)	58	(18.3)
Unknown	8	(1.3)	1	(0.7)	4	(2.3)	3	(0.9)
Metastases, n (%)								
Bone	359	(56.4)	66	(46.5)	91	(51.4)	201	(63.4)
Liver	306	(48.0)	62	(43.7)	77	(43.5)	166	(52.4)
Lung	256	(40.2)	42	(29.6)	72	(40.7)	142	(44.8)
Distal lymph node	189	(29.7)	38	(26.8)	53	(29.9)	98	(30.9)
Regional lymph node	173	(27.2)	30	(21.1)	42	(23.7)	101	(31.9)
Skin	83	(13.0)	16	(11.3)	25	(14.1)	42	(13.2)
Brain	44	(6.9)	10	(7.0)	13	(7.3)	21	(6.6)
Affected side breast	39	(6.1)	6	(4.2)	8	(4.5)	25	(7.9)
Healthy side breast	18	(2.8)	3	(2.1)	3	(1.7)	12	(3.8)
Others	138	(21.7)	31	(21.8)	41	(23.2)	66	(20.8)

*ECOG PS* Eastern Cooperative Oncology Group Performance Status, *ER* estrogen receptor, *PgR* progesterone receptor, *max* maximum, *min* minimum, *SD* standard deviation

^a)^One patient whose number of previous chemotherapy regimens was unknown was included in the analysis

The proportions of patients who started eribulin at 1.4 mg/m^2^ were 79.1%, 88.0%, 79.1%, and 75.1% for all, first-, second-, and third or later-line treatment groups, respectively (Table 2).

**Table 2:** Eribulin treatment status

		All^a)^		First-line		Second-line		Third or later-line	
		*n* = 637		*n* = 142		*n* = 177		*n* = 317	
Start dose (mg/m^2^), *n* (%)	1.4	504	(79.1)	125	(88.0)	140	(79.1)	238	(75.1)
	1.1	91	(14.3)	12	(8.5)	27	(15.3)	52	(16.4)
	0.7	11	(1.7)	2	(1.4)	1	(0.6)	8	(2.5)
	Other	31	(4.9)	3	(2.1)	9	(5.1)	19	(6.0)
Number of cycles	Mean ± SD	7.7 ± 6.8		9.2 ± 8.0		8.0 ± 7.3		6.8 ± 5.8	
	Median (min–max)	5.0	(1–36)	6.5	(1–34)	6.0	(1–36)	5.0	(1–35)
Dose intensity (mg/m^2^/week)	Mean ± SD	0.68 ± 0.18		0.73 ± 0.17		0.69 ± 0.17		0.66 ± 0.18	
	Median (min–max)	0.69	(0.2–0.9)	0.76	(0.3–0.9)	0.7	(0.2–0.9)	0.66	(0.2–0.9)
Relative dose intensity	Mean ± SD	0.73 ± 0.19		0.78 ± 0.19		0.73 ± 0.19		0.70 ± 0.20	
	Median (min–max)	0.74	(0.2–1.0)	0.82	(0.3–1.0)	0.74	(0.2–1.0)	0.71	(0.2–1.0)

*max* maximum, *min* minimum, *SD* standard deviation

^a)^One patient whose number of previous chemotherapy regimens was unknown was included in the analysis

### Effectiveness of using OS

In the effectiveness analysis, 632 of 637 patients were included in the analysis of survival after treatment with eribulin after excluding 5 patients with unknown survival status. In all, first-, second-, and third or later-line treatment groups, the median OS (95% CI) were 15.6 (13.8–17.6), 22.8 (17.3–31.0), 16.3 (12.4–19.9), and 12.6 (11.2–15.1) months, respectively (Table 3).

**Table 3:** Kaplan-Meier estimates for OS and TTF after the treatment with eribulin

	OS				TTF			
		Median, months	1 year, %	2 years, %		Median, months	1 year, %	2 years, %
	*n*	(95% CI)	(95% CI)	(95% CI)	*n*	(95% CI)	(95% CI)	(95% CI)
All^a)^	632	15.6	58.2	35.9	636	4.2	10.4	3.0
		(13.8–17.6)	(54.2–62.1)	(31.9–39.8)		(3.7–4.4)	(8.1–12.9)	(1.9–4.6)
First-line	142	22.8	71.6	48.3	142	5.2	14.1	5.6
		(17.3–31.0)	(63.2–78.4)	(39.6–56.6)		(3.7–5.9)	(9.0–20.3)	(2.6–10.3)
Second-line	175	16.3	58.2	37.0	177	4.2	11.9	2.8
		(12.4–19.9)	(50.3–65.2)	(29.5–44.6)		(3.7–5.1)	(7.6–17.1)	(1.1–6.1)
Third or later-line	314	12.6	52.0	29.5	316	3.8	7.9	2.0
		(11.2–15.1)	(46.1–57.5)	(24.2–34.9)		(3.5–4.2)	(5.3–11.2)	(0.8–4.0)

*CI* confidence interval

^a)^One patient whose number of previous chemotherapy regimens was unknown was included in the analysis. Overall survival (OS) was defined as the time from the first eribulin dose administration until all-cause death. Time to treatment failure (TTF) was defined as the time from the first eribulin dose administration until the date of treatment discontinuation from any cause

Overall, the 1- and 2-year survival rates were 58.2% and 35.9%, respectively (Table 3). In the first-, second-, and third or later-line treatment groups, the 1- vs. 2-year survival rates were 71.6% vs. 48.3%; 58.2% vs. 37.0%; and 52.0% vs. 29.5%, respectively (Table 3).

### Effectiveness using TTF

Excluding 1 patient with an incalculable eribulin administration period, 636 of 637 patients were included in the analysis of TTF. In all, first-, second-, and third or later-line treatment groups, the median TTF (95% CI) were 4.2 (3.7–4.4), 5.2 (3.7–5.9), 4.2 (3.7–5.1), and 3.8 (3.5–4.2) months, respectively (Table 3).

Overall, 10.4% and 3.0% of the patients were estimated to have continued with the eribulin treatment for 1 year and 2 years, respectively (Table 3). In the first-, second-, and third or later-treatment-line, 14.1%, 11.9%, and 7.9% of the patients were estimated to have continued with the eribulin treatment, respectively (Table 3).

### Factors affecting TTF according to multivariate cox regression

**Fig. 1:**
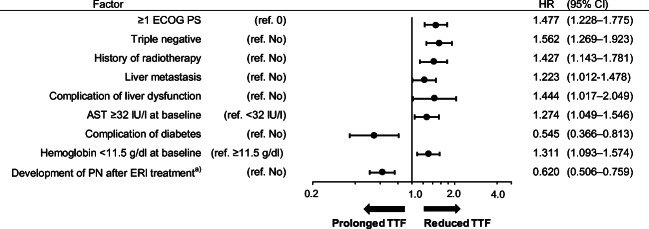
Multivariate Cox regression: factors affecting TTF. ^a)^Development of peripheral neuropathy (PN) after eribulin (ERI) treatment includes new onset of PN and worsening of existing PN from baseline. *AST* Aspartate Aminotransferase, *CI* confidence interval, *ECOG PS* Eastern Cooperative Oncology Group Performance Status, *ERI* eribulin, *HR* hazard ratio, *TTF* time to treatment failure

Multivariate Cox regression analysis was performed to identify the factors influencing TTF. The following factors were significantly associated with the reduced TTF: ≥1 ECOG PS, triple-negative, a history of radiotherapy, liver metastasis, complication of liver dysfunction, AST ≥32 IU/l at baseline, and hemoglobin <11.5 g/dl at baseline. The complication of diabetes and the development of peripheral neuropathy after eribulin treatment was associated with prolonged TTF (Fig. 1).

### Safety

Overall, in the first-, second-, and third or later-line treatment groups; 83.2%, 86.6%, 82.5%, and 82.0% of the patients showed ADRs for all grades, respectively (Table 4).

**Table 4:** All grade ADRs

	All^a)^		First-line		Second-line		Third or later-line	
	*n* = 637		*n* = 142		*n* = 177		*n* = 317	
	*n*	(%)	*n*	(%)	*n*	(%)	*n*	(%)
All grade ADR	530	(83.2)	123	(86.6)	146	(82.5)	260	(82.0)
Neutropenia	373	(58.6)	85	(59.9)	110	(62.1)	177	(55.8)
Leukopenia	358	(56.2)	83	(58.5)	94	(53.1)	180	(56.8)
Peripheral neuropathy	173	(27.2)	44	(31.0)	57	(32.2)	71	(22.4)
Lymphopenia	93	(14.6)	19	(13.4)	25	(14.1)	49	(15.5)
Stomatitis	68	(10.7)	24	(16.9)	19	(10.7)	25	(7.9)
Malaise	64	(10.0)	15	(10.6)	26	(14.7)	23	(7.3)
Aspartate aminotransferase increased	48	(7.5)	11	(7.7)	12	(6.8)	25	(7.9)
Pyrexia	46	(7.2)	5	(3.5)	16	(9.0)	25	(7.9)
Alanine aminotransferase increased	39	(6.1)	11	(7.7)	11	(6.2)	17	(5.4)
Dysgeusia	39	(6.1)	8	(5.6)	11	(6.2)	20	(6.3)
Nausea	36	(5.7)	6	(4.2)	15	(8.5)	15	(4.7)
Anemia	34	(5.3)	9	(6.3)	7	(4.0)	18	(5.7)

Common Terminology Criteria for Adverse Events (version 4.0) all grade adverse drug reactions (ADRs) occurring in ≥5% of the patients in all groups are summarized

^a)^One patient whose number of previous chemotherapy regimens was unknown was included in the analysis

Table 5 summarizes the results of those with grade ≥ 3 ADRs in all, first-, second-, and third or later-line treatment groups. Overall, 61.7% of the patients reported grade ≥ 3 ADRs. In all, first-, second-, and third or later-line treatment groups, the most commonly observed grade ≥ 3 ADRs were neutropenia (49.5%, 54.2%, 48.6%, and 47.6%, respectively); followed by leukopenia (36.6%, 36.6%, 31.6%, and 39.1%, respectively); and lymphopenia (11.8%, 10.6%, 9.6%, and 13.6%, respectively) (Table 5). Other grades ≥3 ADR incidences were below 5.5% in each group (Table 5).

**Table 5:** Grade ≥ 3 ADRs

	All^a)^ *n* = 637		First-line *n* = 142		Second-line *n* = 177		Third or later-line *n* = 317	
	*n*	(%)	*n*	(%)	*n*	(%)	*n*	(%)
Neutropenia	315	(49.5)	77	(54.2)	86	(48.6)	151	(47.6)
Leukopenia	233	(36.6)	52	(36.6)	56	(31.6)	124	(39.1)
Lymphopenia	75	(11.8)	15	(10.6)	17	(9.6)	43	(13.6)
Anemia	16	(2.5)	5	(3.5)	1	(0.6)	10	(3.2)
Gamma-glutamyl transferase increased	18	(2.8)	3	(2.1)	6	(3.4)	9	(2.8)
Peripheral neuropathy	10	(1.6)	3	(2.1)	3	(1.7)	4	(1.3)
Febrile neutropenia	22	(3.5)	2	(1.4)	3	(1.7)	17	(5.4)
Alanine aminotransferase increased	10	(1.6)	2	(1.4)	2	(1.1)	6	(1.9)
Aspartate aminotransferase increased	14	(2.2)	1	(0.7)	2	(1.1)	11	(3.5)
Thrombocytopenia	7	(1.1)	0	(0.0)	2	(1.1)	5	(1.6)

Common Terminology Criteria for Adverse Events (version 4.0) grade ≥ 3 adverse drug reactions (ADRs) occurring in ≥1% of patients in all groups are summarized

^a)^One patient whose number of previous chemotherapy regimens was unknown was included in the analysis

Seventy (11.0%) patients discontinued eribulin treatment due to the ADRs. ADRs occurring in ≥1% of the patients that resulted in eribulin discontinuation were leukopenia (3.0%), neutropenia (2.8%), peripheral sensory neuropathy (2.4%), and malaise (1.3%).

## Discussion

To determine the effectiveness and safety of eribulin as a first- or second-line treatment, we conducted a 2-year post-marketing observational study in patients with HER2-negative advanced breast cancer in a real-world setting, and here, we report the effectiveness and safety of eribulin as a first-, second-, and third or later-line treatment. The median OS and TTF were 15.6 and 4.2 months, respectively.

The OS of 15.6 months was generally similar to those reported in a previous clinical trial (11.3–17.4 months) [10, 18–20]. Moreover, more prolonged OS such as 72.1 months [21] and 22.3 months [22] were reported in real-world studies than in this study. These differences in results may be explained by the differences in the patient’s background characteristics and study designs. One study evaluated OS in patients with estrogen receptor (ER)-positive, HER2-negative metastatic breast cancer in a single institution [21] while in another study, patients were enrolled since 2011 and followed-up until 2015 [22].

In the first-, second-, and third or later-treatment-line groups, OS was 22.8, 16.3, and 12.6 months, respectively. These results according to the treatment line status of eribulin were consistent with previous pre-approval and real-life studies. Previously reported OS results included 16.1 months [13] and 12.4 months [14] for first-line; 21.4 months [12] for the first- or second-line; 10.3 months [14] for the third-line; and 13.1 months [5] for the third or later-lines. However, the results of the phase 2 clinical trial in Japan, which included patients with HER2-negative breast cancer who used eribulin as a first-line treatment, showed a more prolonged OS (35.9 months) than the result of this study [9]. This difference might be attributable to the relatively small number of patients (35 patients) included in the phase 2 study [9]. One phase 3 trial reported similar OS as in this study which was more prolonged in patients using eribulin (16.1 months) than in patients using capecitabine (13.5 months) when these were used as second-line treatment with a manageable safety profile [13]. Another eribulin study in a clinical setting also illustrated a more prolonged OS in an eribulin monotherapy group (22.3 months) than in taxane monotherapy (13.2 months) and taxane plus bevacizumab (12.9 months) groups [22]. Owing to the absence of a comparator in this study, we could not directly compare the OS results by eribulin with other breast cancer drugs. However, because of the beneficial OS results by eribulin as indicated by previous studies, this might be expected in some breast cancer patients in a clinical setting, especially in the initial treatment lines. Indeed, further studies of eribulin, particularly as a first- or second-line treatment are required before drawing any conclusion. Such studies should compare the OS results of eribulin and other breast cancer drugs.

Other results of effectiveness endpoints such as survival rates and TTF were generally consistent with those of previous pre-approval and real-world studies. For instance, in all patients, the 1-year survival rate was reported as 58.2% in this study compared with 64.4% [19] in a previous study. Additionally, the 2-year survival rate was reported as 35.9% in this study compared with 32.8% [19] and 57.2% [23] in previous studies. As for first-line treatment, the 1-year survival rate was reported as 71.6% in this study compared with 65.9% in a previous study [8]. For the third or later-line treatment, the 1-year survival rate was reported as 52.0% in this study compared with 53.9% in a previous study [5]. Furthermore, TTF was reported as 4.2 months in this study compared with 3.91 months [23] and approximately 4 months (127 days) [16] in previous studies on eribulin as a first-, second-, third or later-line treatments. In comparison, 5.2 and 5.3 months [9] were reported in this and a previous study for eribulin as first-line treatment, respectively.

In this study, the complication of diabetes and the development of peripheral neuropathy after eribulin treatment were associated with prolonged TTF in multivariate Cox regression analysis. However, factors associated with reduced TTF included low hemoglobin levels at baseline (<11.5 g/dl). Interestingly, development of peripheral neuropathy after eribulin treatment was associated with prolonged TTF in this study. Additionally, a paclitaxel study demonstrated that early occurrence of peripheral neuropathy may be a positive prognostic indicator for TTF [24]. This paclitaxel study suggested that this may be explainable by the dose of paclitaxel, as occurrence of peripheral neuropathy is dependent on dose [24]. Therefore, the higher drug concentrations lead to higher incidence of neuropathy events as well as better efficacy [24]. This partly corroborates our study results that the first-line and second-line treatment groups were treated with relatively high start dose of eribulin (1.4 mg/m^2^), dose intensity, and relative dose intensity. In this study, low hemoglobin levels at baseline were associated with reduced TTF. Moreover, previous studies associated the low hemoglobin levels including anemia with poor survival prognosis in breast cancer patients [25–28]. Thus, low hemoglobin levels may be a robust prognostic indicator for reduced TTF for breast cancer patients. Further investigation of factors affecting TTF by eribulin will advance breast cancer treatment.

Irrespective of the patients’ treatment line, the incidence of grade ≥ 3 ADRs did not largely differ, and hematologic events (neutropenia, leukopenia, and lymphopenia) were the most commonly observed grade ≥ 3 ADRs. The remaining ADRs had a relatively low incidence of <5.5%. Overall, approximately 10% of the patients discontinued eribulin due to ADRs. These findings are generally consistent with those of previous pre-approval and post-marketing studies. These included studies on eribulin as a first- or second-line treatment, although direct comparisons are limited by study design differences (e.g. the majority of the studies assessed eribulin safety by adverse events) [9, 10, 15, 16, 18, 19]. Hence, eribulin was well-tolerated, as shown in previous studies, although ADRs, such as the hematologic events, should be taken into consideration for eribulin administration, regardless of the patients’ eribulin treatment line status.

Guidelines in Japan recommend the sequential administration of a single agent to maintain or improve the QoL for metastatic or recurrent breast cancer except in extreme cases [29]. Since the goal of metastatic breast cancer treatment is to maintain or improve survival and QoL while alleviating adverse symptoms, a single agent that can address all of those is required. As discussed above, we confirmed a well-tolerated safety profile of eribulin in all groups, consistent with previous clinical and post-marketing studies. This study did not assess QoL. However, a previous eribulin study demonstrated that eribulin seemed to maintain health-related QoL in almost all the patients in the study [12]. Therefore, eribulin might represent another first-line treatment candidate for HER2-negative metastatic or recurrent breast cancer, similar to commonly selected drugs (anthracyclines and taxanes). Further studies on eribulin as first-line treatment to assess effectiveness, safety, and QoL in a real-life setting are warranted.

Interpretation of this study results may require careful consideration. First, we only assessed the effectiveness and safety of eribulin in patients with HER2-negative breast cancer. Therefore, our study results are not applicable to patients with HER2-positive breast cancer. Second, our OS results were limited to a maximum of 2-year follow-up. Thirdly, OS is generally affected by the post-treatment period after completion of chemotherapy and by the time taken to complete a previous clinical trial because new breast cancer treatments could have emerged. Thus, comparisons of OS results with that of previous clinical trials may be limited.

In conclusion, the effectiveness and safety results of eribulin as a first- or second-line treatment in a clinical setting were favorable and in line with previous pre-approval clinical and post-marketing study results. Therefore, these results suggest that eribulin may be an additional candidate for first-line treatment of patients with HER2-negative advanced breast cancer in Japan.
